# Interaction of Sleep and Cortical Structural Maintenance From an Individual Person Microlongitudinal Perspective and Implications for Precision Medicine Research

**DOI:** 10.3389/fnins.2020.00769

**Published:** 2020-07-31

**Authors:** John Wall, Hong Xie, Xin Wang

**Affiliations:** ^1^Department of Neurosciences, University of Toledo College of Medicine and Life Sciences, Toledo, OH, United States; ^2^Department of Psychiatry, University of Toledo College of Medicine and Life Sciences, Toledo, OH, United States; ^3^Department of Radiology, University of Toledo College of Medicine and Life Sciences, Toledo, OH, United States

**Keywords:** circadian/allostatic/homeostatic, glymphatic, human brain maintenance, *n*-of-1 analyses, precision medicine research, sleep, synaptic homeostasis

## Abstract

Sleep and maintenance of brain structure are essential for the continuity of a person’s cognitive/mental health. Interestingly, whether normal structural maintenance of the brain and sleep continuously interact in some way over day–week–month times has never been assessed at an individual-person level. This study used unconventional microlongitudinal sampling, structural magnetic resonance imaging, and *n*-of-1 analyses to assess normal interactions between fluctuations in the structural maintenance of cerebral cortical thickness and sleep duration for day, week, and multi-week intervals over a 6-month period in a healthy adult man. Correlation and time series analyses provided indications of “if–then,” i.e., “if” this preceded “then” this followed, sleep-to-thickness maintenance and thickness maintenance-to-sleep bidirectional inverse interactions. Inverse interaction patterns were characterized by concepts of graded influences across nights, bilaterally positive relationships, continuity across successive weeks, and longer delayed/prolonged effects in the thickness maintenance-to-sleep than sleep-to-thickness maintenance direction. These interactions are proposed to involve normal circadian/allostatic/homeostatic mechanisms that continuously influence, and are influenced by, cortical substrate remodeling/turnover and sleep/wake cycle. Understanding interactions of individual person “-omics” is becoming a central interest in precision medicine research. The present *n*-of-1 findings contribute to this interest and have implications for precision medicine research use of a person’s cortical structural and sleep “-omics” to optimize the continuous maintenance of that individual’s cortical structure, sleep, and cognitive/mental health.

## Introduction

There is growing interest in precision medicine research into how to optimize brain and body maintenance at an individual-person level (McEwen and Getz, 2013; Collins and Varmus, 2015; Insel and Cuthbert, 2015; Cole, 2018; Goetz and Schork, 2018; Huang and Hood, 2019). This has led to unconventional work using microlongitudinal time series measures and biographical metrics or “-omics” that are analyzed at an *n*-of-1, rather than group, level. Differing from macrolongitudinal approaches that focus on age or aging across the lifespan, microlongitudinal analyses track fluctuations in measures at short intervals over substantial time scales to assess continuously ongoing interaction dynamics.

Provocative new insights have been defined from “-omics” analyses of, e.g., genome, transcriptome, proteome, metabolome, microbiome, physiome, virome, and exposome measures from the body (David et al., 2014; Li et al., 2017; Chen et al., 2018; Jiang et al., 2018; Zhou et al., 2019). With respect to the brain, MRI analyses have focused on brain function (Choe et al., 2015; Laumann et al., 2015; Poldrack et al., 2015; Braga and Buckner, 2017; Gordon et al., 2017; Donnelly-Kehoe et al., 2019), and to a lesser degree, structure at subcortical (Maclaren et al., 2014) or cortical (Barth et al., 2016; Filevich et al., 2017; Wall et al., 2017; Xie et al., 2018) levels. Further *n*-of-1 “-omics” research is likely forthcoming and justified.

With regard to the latter cortical structure work, we recently reported microlongitudinal MRI time series data from a healthy adult man which suggested that the hemispheric thicknesses of his cerebral cortex were not statically maintained from week–week as predicted from age and aging studies of normal adult groups (Wall et al., 2017). Ongoing maintenance instead involved week–week reversing incremental and decremental thickness fluctuations that appeared to reflect normal continuous remodeling/turnover of cortical substrates. This suggested a hypothesis of adult cortical maintenance whereby structural stability is maintained by continuously ongoing thickness fluctuation, as opposed to static preservation of thickness previously formed during development. This maintenance-related fluctuation in cortical structure potentially resembles maintenance of the body, where the structural stability of tissues, organs, and systems entails continuous fluctuation due to the normal dynamic regulatory actions of circadian, allostatic, and homeostatic mechanisms (Ganzel et al., 2010; Juster et al., 2010; McEwen and Karatsoreos, 2015). This raises the interesting possibility that normal fluctuations in thickness maintenance of the cortical part of his brain may have been interacting with factors that affect the fluctuations in broader maintenance of his body.

As one such factor, sleep–wake cycle affects combined circadian/allostatic/homeostatic controls on molecular, cellular, system, and behavioral processes that contribute to continuous body maintenance (Carroll et al., 2015; Musiek and Holtzman, 2016; Jones et al., 2019). Both sleep–wake cycle and cortical thickness are individual specific. Interestingly, how or even whether normal fluctuations in an individual’s sleep and cortical thickness maintenance continuously interact is not understood.

The present investigation explored this issue with an unconventional approach that differed in the following ways from existing MRI studies of cortical thickness and sleep. First, most existing studies are based on limited thickness sampling in each individual, obtained with a single cross-sectional scan or two or a few macrolongitudinal-design scans which are aimed at defining concepts that apply to groups. In contrast, the present study is based on microlongitudinal *n*-of-1 analyses of extensive thickness sampling over time in an individual, aimed at defining concepts that apply to an individual. Second, much MRI work focuses on how abnormal sleep or sleep deprivation relate to cortical thickness. In contrast, we focused on whether ongoing normal fluctuations in sleep might be related to cortical thickness. Third, sleep is commonly assumed to have a comparable influence on both sides of the brain, including the left and the right cortices. This bilateral assumption has not been directly tested with respect to normal maintenance at the individual brain level. Our previous microlongitudinal analyses in the above individual suggested that the maintenance of thickness of his two cortices was asymmetric in some respects (Xie et al., 2018). Consistent with common assumptions, the present study assessed relationships between sleep and thickness maintenance for both cortices considered together; however, given these previous findings in this individual, we further assessed sleep relationships with each cortex separately to examine possible side–side laterality differences. Finally, most existing MRI analyses retrospectively identify non-directional correlations between sleep and cortical thickness. In contrast, the present microlongitudinal time series analyses provided a prospective examination of short-interval temporal relationships between preceding sleep and subsequent thickness maintenance and between preceding thickness maintenance and subsequent sleep. This provided tests of “if–then,” i.e., “if” this preceded “then” this followed, direction(s) of relationships.

Using data from the microlongitudinal time series in our above studied individual, where cortical thickness maintenance was repeatedly assessed at weekly intervals for 6 months, three questions were addressed: (1) Are fluctuations in sleep duration at earlier times related to maintenance of cortical thickness at later times? (2) Are fluctuations in maintenance of cortical thickness at earlier times related to sleep duration at later times? (3) Do cortical thickness maintenance and sleep duration interact bidirectionally over short intervals?

Sleep duration was studied because it is integrated with circadian/allostatic/homeostatic mechanisms that affect brain health. To deal with the current lack of knowledge about what sleep time scales may potentially be involved in short-interval relationships with thickness maintenance, we assessed sleep durations for week, week segment, and individual night time windows.

Thickness was used as an index of cortical structural maintenance because it can be objectively and repeatedly measured in an individual person with automated programs (Fischl, 2012) and because it is a sensitive indicator of differences in cortical structural integrity of healthy adults vs. adults with cortical disorders (Suh et al., 2016; Macey et al., 2018; van Erp et al., 2018; Ye et al., 2020). Cortical structural, including thickness, maintenance is a continuously ongoing process. This is evident from the fact that loss of blood and related glucose and oxygen flow to any cortical location at any time results in rapid structural maintenance failure and deterioration that begins within minutes. Hemispheric mean thickness was used as a measure because it was considered appropriate to assess the spatially ubiquitous nature of continuously ongoing structural maintenance across the hemisphere.

The issue and questions that we addressed have been overlooked. However, we felt that (1) their significance for understanding relationships between cortical structural maintenance and sleep at an individual level and (2) the availability of the necessary but rare microlongitudinal cortical and sleep measures from the same individual, justified the present investigation. We took a non-hypothesis-driven approach to attempt to identify individual-based concepts that may differ from hypotheses suggested by existing group-based work.

The results suggest concepts of normal interaction between ongoing maintenance of cortical structure and sleep from an individual-person perspective that are not recognized and that have precision medicine research implications.

## Materials and Methods

### The Individual’s Health and Sleep Habits

#### Health

The subject is a left-handed 66-year-old man who was selected based on his motivated willingness to undergo weekly MRI scanning and daily sleep and health monitoring over the 6-month study period. As indicated below, an additional reason was that he had a good health history.

He has been active across life (e.g., regular bicycling, jogging) and had a mean (SD) activity level of 10,714 (± 3,260) steps/day over the study. He has been a vegetarian since 2000 and has not used tobacco since 1980 or alcohol since 2000. Prior to those times, he was a sporadic pipe smoker during the 1970s and a minimal alcohol consumer. He had not required or taken regular medications and never used recreational drugs. From direct knowledge and self-report, he has no history of chronic medical problems, childhood abuse, psychiatric illness, concussion, or head trauma, and the MRI scans indicated no brain abnormalities. He experienced no illnesses or trauma during the study, and day-to-day activities involved usual work and home routines with no travel, training, medical, or other unusual interventions. These activities were considered consistent with usual daily maintenance of brain and body.

Daily health monitoring over the study included previously reported measures of, e.g., pulse, blood pressure, blood glucose, oral temperature, and weight and, at the end of the study, waist circumference, hemoglobin A_1__*c*_ (HbA_1__*c*_), lipid, and other measures (Wall et al., 2017). All measures were entered into a database at the time of measure to preclude recall error and were not further examined until after study completion. At that time, three physicians who were not involved in the study independently reviewed these measures and rated all to be within or approximate healthy ranges [marginally low pulse (57 ± 3 bpm) and marginally high systolic pressure (124 ± 7 mmHg) upon arising in mornings].

His measures were further compared to threshold criteria for nine biomarkers which, collectively, have been used to define allostatic load (Parente et al., 2013; Chen et al., 2014). The mean measures taken daily over the study did not reach thresholds for high allostatic load for pulse (57 bpm, threshold ≥ 90 bpm), systolic blood pressure (124 mmHg, threshold ≥ 140 mmHg) and diastolic blood pressure (79 mmHg, threshold ≥ 90 mmHg). In addition, further measures taken at the end of the study did not reach high allostatic load thresholds for total cholesterol (174 mg/dl, threshold ≥ 240 mg/dl), high-density lipoprotein cholesterol (HDL, 53 mg/dl, threshold ≤ 40–50 mg/dl), glycosylated hemoglobin (HbA1c, 5.4%, threshold ≥ 6.4%), albumin (4.2 g/dl, threshold < 3.8–4.0 g/dl), C-reactive protein (1.7 mg/L, threshold > 3 mg/L), and body mass index (21–22 kg/m^2^, threshold ≥ 30 kg/m^2^). Collectively, these measures are consistent with low allostatic load.

Metabolic syndrome was assessed using standard criteria (i.e., ≥ 3 measures above cut-point criteria for waist circumference, triglycerides, high-density lipoprotein, blood pressure, and fasting glucose) (Alberti et al., 2009) and found not to be a risk. Transcranial Doppler ultrasound measures taken at the end of the study by an experienced sonographer (Neurovascular Lab, Cleveland Clinic) indicated that blood flow velocities for cortical arteries were within normal ranges.

Responses to the Beck Anxiety Inventory (BAI), Beck Depression Inventory (BDI-II), and Oxford Happiness Questionnaire (OHQ), all modified to reference that day, were recorded each evening. The mean (SD) daily scores indicated that he was within normal anxiety [BAI: 0.01 (0.108)], no depression [BDI-II: 0.04 (0.200)], and happy [OHQ: 5.18 (0.10)] ranges. The mean (SD) daily responses to two OHQ items, which rated health states on a scale of 1–6, indicated that the subject felt mentally alert [item #21: “I feel fully mentally alert”: 5.15 (0.354) with 6 = strongest agreement with statement] and healthy [item #28: “I don’t feel particularly healthy”: 5.99 (0.076), item reverse-scored with 6 = strongest disagreement with statement].

#### Sleep Habits

Before and during the study, the individual worked a consistent weekday (Monday–Friday, ≈08:00–18:00) and weekend (Saturday–Sunday, ≈2–4 h/day) schedule. He did not cross time zones over the several months prior to or during the study. He regularly followed practices that promoted healthy sleep, including no TV, mobile phones, or computers/monitors in the bedroom, no use of electronic devices ≤ 0.5 h before retiring, and no use of caffeine-containing products in the evenings. During the study, he maintained his pre-study to-bed and rise times and regular attempt to get 7–8 h of sleep/night. On week work mornings, he typically woke up with an alarm and on weekend “free” mornings without an alarm. From self-report and spouse confirmation, he did not have difficulty initiating sleep, usually fell asleep within ≈10 min after going to bed, slept until waking the following morning, and did not suffer from sleep-related disorders [insomnia, apnea (snoring, choking), restless leg syndrome, sleep walking, nocturia, or narcolepsy]. He did not nap during the day.

### Microlongitudinal Design

The microlongitudinal time series design used to repetitively measure the maintenance of mean hemispheric thickness of each cortex has been described previously (Wall et al., 2017). Briefly, MRI scans were made on 22 dates across a 25-week period that spanned late summer, fall, and early winter seasons. Except for missed scans at weeks 2, 6, and 7, scans were taken at 1-week intervals on Sundays around the same mid-day start time (mean ± SD: 13:55 ± 2.1 h). On each date, two scans were completed in one session, with removal from the scanner between the first scan (scan A) and the second scan (scan B) (≈5 min between scans). This provided 44 measures of thickness maintenance over a total sample time of 8.2 h.

Sleep durations were measured every night over the scan period. Sleep durations during weeks before and after the scan period were also included to assess temporal relationships between sleep that preceded the first scan and that followed the last scan.

### MRI Scans, Scan Processing, and Thickness Measures

MRI scan and scan processing protocols, as well as applied stringent controls to quantify and reduce measurement error, have been previously described (Wall et al., 2017). Briefly, T1-weighted scans of the entire brain were made with a 3T GE Signa scanner (164 continuous axial slices, voxel size 1 mm × 1 mm × 1 mm). All scans were made with the same scanner, head coil, and scan parameters. During the study, regular scanner quality assurance tests identified no problems and scanner upgrades were not done.

Image processing, done with automated FreeSurfer procedures^1^, has been previously described (Wall et al., 2017). To treat data from all scans as equal and independent measures, each scan was processed individually without cross-scan registration or averaging. Thickness measures were taken in native space without transformation to a template.

Cortical thickness was defined at ≈150,000 vertex locations/hemisphere, and mean hemispheric cortical thickness (mm) was determined for each hemisphere using all vertex measures from that hemisphere. To ensure uniform processing, all scans were processed at one time after the study period using one workstation, operating system, and FreeSurfer program.

### Sleep Measures

Sleep onset and end times were entered into a database each morning upon arising to preclude recall error. Sleep onset times were distinguished from preceding to-bed times, and sleep end times were distinguished from rise times to provide a closer delineation of sleep durations. Nightly sleep duration (h) was defined as sleep end time minus sleep onset time. Mean sleep onset, end, and mid-sleep (halfway between onset and end) times were used to identify chronotype.

### Correlation Analyses

Initial assessments focused on relationships, first, between preceding sleep duration vs. subsequent thickness maintenance and, second, in the reverse direction between preceding thickness maintenance vs. subsequent sleep duration.

No previous attempts have been made to study correlations between sleep duration and cortical thickness maintenance at repeated short intervals for an extended period in an individual person. Uncertainties about possibilities that sleep duration for a particular night or accumulated effects of sleep durations over some number of nights might be related to thickness maintenance was an issue of consideration for the design of analyses in both directions. To provide an *a priori* systematic plan of study, a strategy was applied that first analyzed accumulated sleep durations over longer time windows, which was followed by analyses for shorter windows to give resolution for a range of time scales. Since thickness measures were taken at weekly intervals on Sundays, initial correlations in both directions correspondingly focused on accumulated sleep duration for Sunday–Saturday night week periods that preceded and followed Sunday thickness measures. These comparisons subsequently led to further analyses of week segment and individual night periods that further bracketed effects.

Given the current assumptions that sleep has similar effects on the left and the right cortices, the initial analyses used pooled left and right thickness maintenance measures. Further analyses considered the left and the right cortices separately to test if relationships were similar or different for the two hemispheres.

Data were plotted in scatterplots with associated linear regression lines and tested with bivariate correlation tests (Pearson *r*). These correlation analyses provided initial tests of contingent “if–then,” i.e., “if” earlier “then” later, unidirectional relationships between preceding sleep and subsequent thickness maintenance and between preceding thickness maintenance and subsequent sleep.

### Time Series Analyses

The correlation analyses suggested that there were unidirectional inverse relationships between preceding sleep and subsequent thickness maintenance and between preceding thickness maintenance and subsequent sleep. Time series analyses were subsequently used to firstly confirm findings of unidirectional inverse relationships. Moreover, time series analyses examined the temporal patterns of these relationships, e.g., times and incidences of occurrence across the study, and whether unidirectional inverse relationships occurred at individual times as isolated events and/or across successive times as bidirectional interactions. Time series analyses used the following thinking and procedures.

The inverse relationships seen in the correlation analyses signified that *higher* levels of the preceding variable related to *lower* levels of the subsequent variable and, conversely, *lower* levels of the preceding variable related to *higher* levels of the subsequent variable. To assess sequences of inverse relationships, higher vs. lower sleep durations and thickness maintenances were distinguished by expressing measures as percentiles. Specifically, sleep durations were converted to percentiles that indicated the percentile rank of each sleep duration measure during the study, with percentiles above and below the median 50th percentile reflecting respectively higher and lower sleep durations. Similarly, thickness maintenance measures were converted to percentiles indicating the percentile rank of each thickness maintenance measure during the study, with percentiles above and below the median 50th percentile reflecting respectively higher and lower thickness maintenances.

Sleep and thickness maintenance percentiles were plotted together as a function of when they occurred during the study, thus permitting the identification of higher-to-lower and lower-to-higher inverse relationships in sleep-to-thickness and thickness-to-sleep sequences over successive times. This permitted the identification of (a) unidirectional preceding sleep to subsequent thickness maintenance inverse relationships, (b) unidirectional preceding thickness maintenance to subsequent sleep inverse relationships, and (c) progressions of bidirectional inverse interactions resulting from successive sequences of (a) and (b) relationships. Incidences of associations that did not fit the 50th percentile criteria were further identified. Separate time series analyses were done using sleep durations for week and individual night periods.

### Significance Levels

Statistical analyses and plots of data were done with SPSS. An initial two-tailed significance level of *p* ≤ 0.05 was used for the tests. Individual analyses involved one to four tests. For analyses that involved multiple tests, Bonferroni-corrected levels of *p* = 0.05/number of tests were applied to correct for multiple tests and reduce the chances of false positive results. Results where *p* ≤ Bonferroni level were considered as significant, whereas results where *p* > Bonferroni level but ≤ 0.05 were considered as trends for significance.

### Blind Controls

Controls were used to ensure double-blinded tests of relationships: (1) Thickness measures were not defined until after the study period and all scanning had been completed. (2) Similarly, sleep durations were not defined until after the study period. (3) Thickness and sleep duration measures were defined independently by different investigators, who were blind to the other measure, and not altered in subsequent analyses.

## Results

### Cortical Thickness Maintenance Measures

**FIGURE 1:**
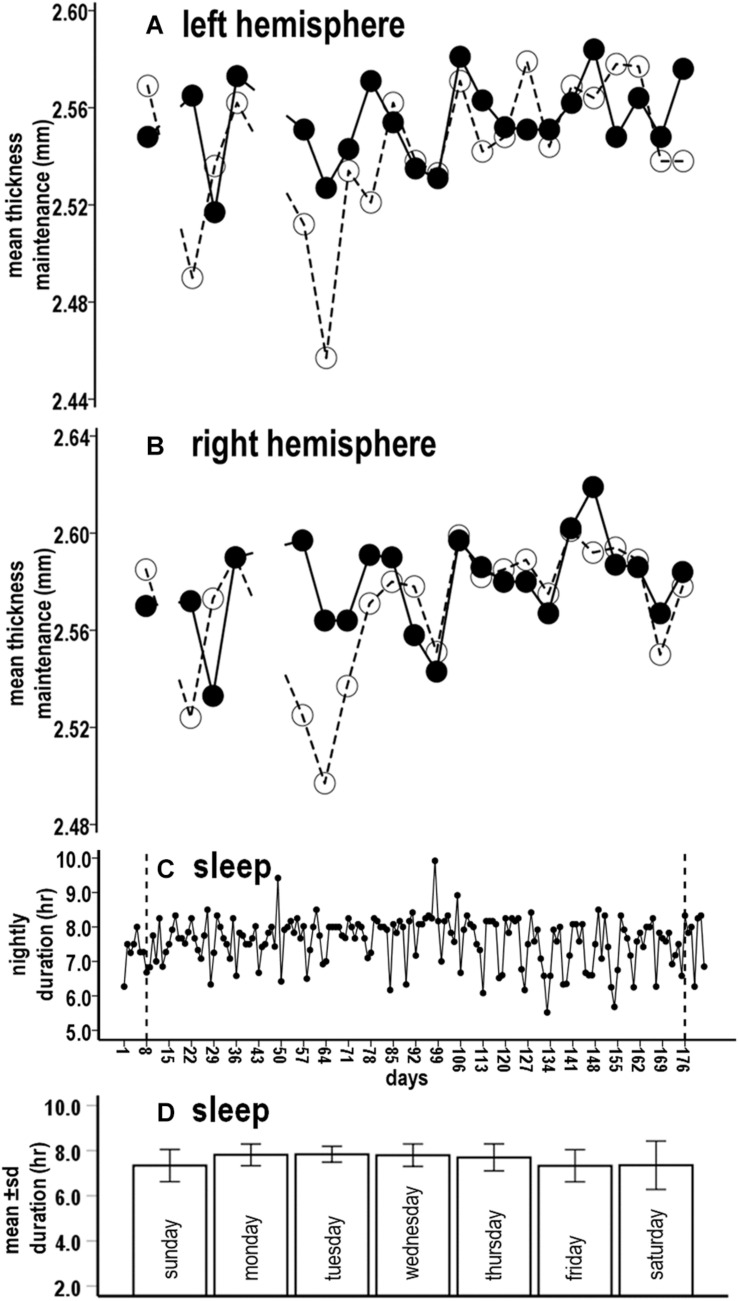
Concurrent microlongitudinal measures of left **(A)** and right **(B)** hemisphere mean thickness maintenances and nightly sleep durations **(C)** across the study. In **(A,B)**, solid lines indicate scan A and dashed lines indicate scan B. Breaks in the lines indicate missing scan data at weeks 2, 6, and 7. Study period time indicated in **(C)** also applies to **(A,B)**, with the vertical lines in **(C)** corresponding to first and last scan days in **(A,B)**. Mean (± SD) sleep durations for nights of each day are indicated in **(D)**.

Previously reported mean thickness maintenance measures for each hemisphere are plotted for the study period (Figures 1A,B; Wall et al., 2017). The thickness of each hemisphere underwent reversing incremental and decremental fluctuations from week to week that were not attributable to measurement error (Wall et al., 2017). The maintenance of the right hemisphere thickness was consistently larger than the maintenance of the left hemisphere thickness, with right and left mean thickness maintenances of 2.574 and 2.549 mm, respectively (Wall et al., 2017). Runs tests previously indicated that the thickness maintenance of each hemisphere did not progressively change over the study (Wall et al., 2017).

### Sleep Measures and Ratings

Nightly sleep durations fluctuated around an overall mean of 7.6 h between minimal and maximal durations of 5.5 and 9.9 h (Figure 1C). The distribution of mean sleep durations across nights of the week did not differ from a null horizontal distribution and standard deviations highly overlapped, thus indicating similar durations across nights (Figure 1D, Kolmogorov–Smirnov distribution test, *p* = 0.239). Sleep periods were shifted later by ≈1 h on weekend “free” nights. More specifically, respective mean (SD) sleep onset, end, and mid-sleep times for week work nights were 22:24 (0:32), 6:06 (0:30), and 2:15 (0:26) and for weekend “free” nights were 23:32 (0:35), 6:52 (0:45), and 3:12 (0:30). These times appear consistent with an early-intermediate chronotype (Fischer et al., 2017).

With further respect to sleep regularity, sleep durations for almost all nights (98%, 178/182) ranged between > 6 to < 9 h/night and for the large majority of nights (80%, 145/182) between 7 and 9 h/night. A runs test of nightly sleep durations was not significant (*p* = 0.301), thus suggesting that sleep duration did not progressively change over the study.

Mean (SD) daily responses to sleep relevant items #5 and #25 on the OHQ suggested that he regularly woke up rested and had a great deal of energy: #5 – “I rarely wake up feeling rested” [5.98 (0.132), item reverse-scored with 6 = strongest disagreement with statement] and #25 – “I feel I have a great deal of energy” [4.97 (0.253), with 6 = strongest agreement with statement]. Mean (SD) daily responses to sleep-relevant items on the BDI-II suggested that he did not experience: #15 – loss of energy” [0.05 (0.219)], #16 – “changes in sleeping pattern” [0 (0.0)], #19 – “concentration difficulty” [0 (0.0)], or #20 – “tiredness or fatigue” [0.01 (0.074)] (each item rated 0–3, with 0 indicating non-occurrence), respectively. These ratings suggest that sleep quality was consistently good.

### Question 1: Are Fluctuations in Sleep Duration at Earlier Times Related to Maintenance of Cortical Thickness at Later Times?

#### Analysis 1. Sleep Duration Over the Preceding Week vs. Subsequent Thickness Maintenance

**FIGURE 2:**
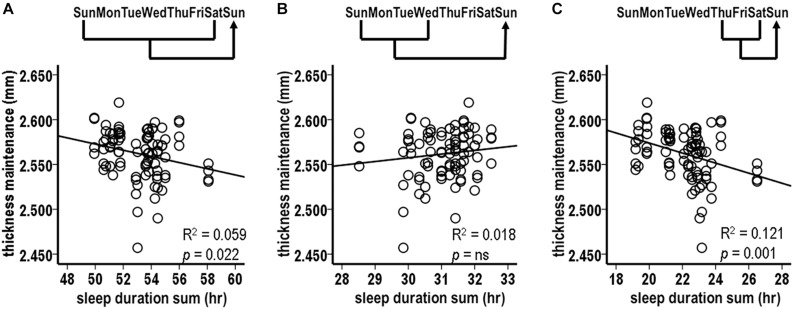
Relationships between preceding sleep durations and subsequent thickness maintenances from tests comparing accumulated sleep durations over preceding Sunday-Saturday week **(A)**, Sunday-Wednesday early week segment **(B)**, and Thursday-Saturday latter week segment **(C)** periods vs. thickness maintenances on subsequent Sundays. Fluctuations in sleep durations over preceding week **(A)** and latter week segment **(C)** periods were significantly inversely related to subsequent thickness maintenance.

Sampling of thickness maintenance was done mid-day on Sundays at weekly intervals. Corresponding to this weekly sampling, the initial analysis examined the relation between fluctuations in accumulated sleep hours over the preceding Sunday–Saturday night week periods vs. thickness maintenances for the subsequent Sunday. There was a significant inverse relationship between preceding week sleep duration sums and subsequent thickness maintenances (Figure 2A; *R*^2^ = 0.059, *p* = 0.022).

Given this relationship, a *post hoc* test was done for the same Sunday thickness maintenances vs. sleep duration sums for the next earlier week. This was not significant (*R*^2^ = 0.012, *p* = 0.300) and led to focusing on sleep within the week that preceded the thickness measures.

#### Analysis 2. Sleep Durations Over Preceding Week Segments vs. Subsequent Thickness Maintenance

The above results suggested the merit of examining the effects of accumulated sleep durations for different segments of the preceding week. Analysis 2 examined the relationships for sums of sleep hours for Sunday–Wednesday and for Thursday–Saturday night segments.

The accumulated sleep hours for preceding Sunday–Wednesday nights were not significantly related to fluctuations in subsequent Sunday thickness maintenances (Figure 2B, *R*^2^ = 0.018, *p* = 0.214). In contrast, the accumulated hours for the preceding Thursday–Saturday nights had a significant inverse relationship with fluctuations in subsequent thickness maintenances (Figure 2C, *R*^2^ = 0.121, *p* = 0.001). Consistent with the lack of relationship for the Sunday–Wednesday segments, this relationship had a steeper regression line slope than the relationship for the entire week (*R*^2^ = 0.121 vs. 0.059, respectively). This suggested a gradient in influence, with this latter week segment having a stronger influence than the preceding week as a whole.

#### Analysis 3. Sleep Durations Over Preceding Individual Nights vs. Subsequent Thickness Maintenance

Given the findings for the Thursday–Saturday week segment, relationships were tested between sleep durations for the preceding individual Thursday, Friday, and Saturday nights and thickness maintenance on the subsequent Sunday.

**FIGURE 3:**
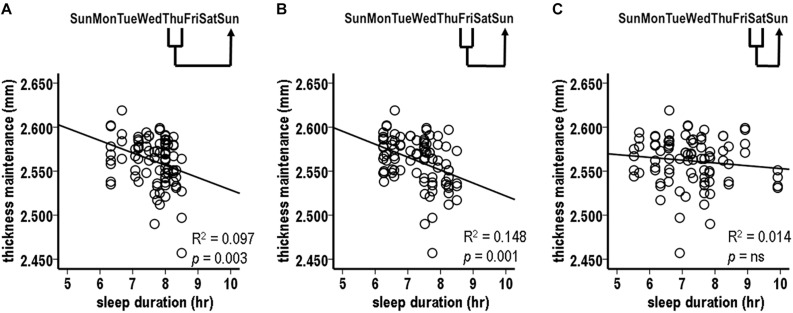
Relationships between preceding sleep durations and subsequent thickness maintenances from tests comparing sleep durations for preceding **(A)** Thursday, **(B)** Friday, and **(C)** Saturday individual nights vs. thickness maintenances on subsequent Sundays. Fluctuations in sleep durations for preceding Thursday **(A)** and Friday **(B)** nights were significantly inversely related to subsequent thickness maintenance.

The sleep durations for Thursday and Friday nights, i.e., the preceding 3rd and 2nd nights, were each significantly inversely related to subsequent thickness maintenance (Figures 3A,B; Thursday: *R*^2^ = 0.097, *p* = 0.003; Friday: *R*^2^ = 0.148, *p* < 0.001). In contrast, the sleep durations for the preceding Saturday nights were not significantly related to subsequent thickness maintenance (Figure 3C; *R*^2^ = 0.014, *p* = 0.265). *Post hoc* individual night tests for the remaining Sunday–Wednesday nights were also not significant. These results suggest a gradient in strength of the preceding night sleep effects on subsequent thickness maintenance, with stronger effects prolonged/delayed over 2–3 days.

#### Analysis 4. Laterality Analysis of Sleep Duration Over Preceding Individual Nights vs. Subsequent Thickness Maintenances for Separate Left and Right Hemispheres

Analysis 3 results from Thursday and Friday nights, with strongest relationships, used pooled thickness maintenances from both hemispheres. To test if the fluctuations in sleep durations for these nights were related to subsequent thickness maintenances of each hemisphere, thickness maintenances of the right and the left hemispheres were considered separately.

**FIGURE 4:**
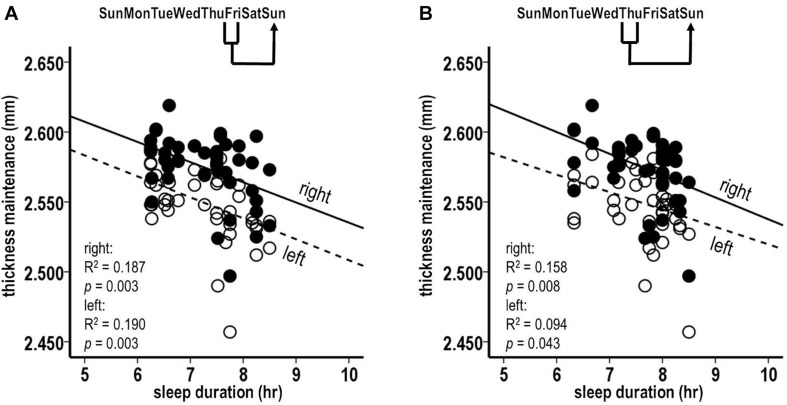
Laterality analyses of fluctuations in sleep durations for preceding Friday **(A)** and Thursday **(B)** nights vs. thickness maintenances for separate right (filled circles, solid regression lines) and left (unfilled circles, dashed regression lines) hemispheres on subsequent Sundays. Fluctuations in sleep durations for preceding Friday nights were significantly inversely related to subsequent thickness maintenances of the right and left hemispheres **(A)**. Fluctuations in sleep durations for preceding Thursday nights were significantly inversely related to subsequent thickness maintenances of the right hemisphere, and had an inverse relationship trend to subsequent thickness maintenances of the left hemisphere **(B)**.

The sleep durations for the preceding Friday night were significantly inversely related to the subsequent thickness maintenances of both the right and the left hemispheres (Figure 4A; right, *R*^2^ = 0.187, *p* = 0.003; left, *R*^2^ = 0.190, *p* = 0.003). The sleep durations for the preceding Thursday night were significantly inversely related to the subsequent thickness maintenance of the right hemisphere and had an inverse relationship trend to the maintenance of the left hemisphere (Figure 4B; right, *R*^2^ = 0.158, *p* = 0.008; left, *R*^2^ = 0.094, *p* = 0.043). These results provided indications that the preceding stronger sleep influences had subsequent bilateral inverse effects on thickness maintenance.

### Question 2: Are Fluctuations in Maintenance of Cortical Thickness at Earlier Times Related to Sleep Duration at Later Times?

The correlation analyses next shifted to test relationships in the opposite direction, i.e., whether fluctuations in preceding cortical thickness maintenance were related to subsequent sleep durations.

#### Analysis 5. Preceding Thickness Maintenance vs. Sleep Duration Over the Subsequent Week

**FIGURE 5:**
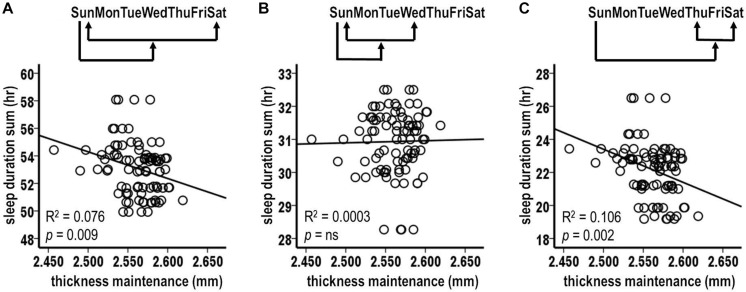
Relationships between preceding thickness maintenances and subsequent week sleep durations from tests comparing mid-day thickness maintenances on preceding Sundays vs. accumulated sleep durations over subsequent Sunday-Saturday week **(A)**, Sunday–Wednesday early week segment **(B)**, and Thursday-Saturday latter week segment **(C)** periods. Fluctuations in preceding thickness maintenance were significantly inversely related to summed sleep durations for subsequent Sunday-Saturday week **(A)** and Thursday-Saturday latter week segment **(C)** periods. Note that compared to Figures 2–4, the axes in these scatterplots and Figure 6 are inverted to reflect that sleep duration was the preceding variable in Figures 2–4, whereas here and in Figure 6 thickness maintenance is the preceding variable.

There was a significant inverse relationship between thickness maintenances on the preceding Sunday and the accumulated sleep hours over the subsequent first Sunday–Saturday week period (Figure 5A; *R*^2^ = 0.076, *p* = 0.009). A further analysis indicated that an inverse relationship did not extend into the nights of the following second week (*R*^2^ = 0.030, *p* = 0.105). This led to a focus on the effects of thickness maintenance on sleep durations during the subsequent first week.

#### Analysis 6. Preceding Thickness Maintenance vs. Sleep Duration Over Segments of the Subsequent Week

The analyses of preceding Sunday thickness maintenances vs. accumulated sleep hours over the early and the latter segments of the subsequent week indicated no significant relationship with sleep duration sums for the early Sunday–Wednesday segment (Figure 5B; *R*^2^ = 0.0003, *p* = 0.866). In contrast, there was a significant inverse relation for the latter Thursday–Saturday week segment (Figure 5C; *R*^2^ = 0.106, *p* = 0.002). This latter week relationship had a steeper regression line slope than the relationship for the subsequent entire week (*R*^2^ = 0.106 vs. 0.076, respectively), thus suggesting a gradient in influence with the latter week segment receiving a stronger influence than the week as a whole.

#### Analysis 7. Preceding Thickness Maintenance vs. Sleep Duration for Subsequent Individual Nights

**FIGURE 6:**
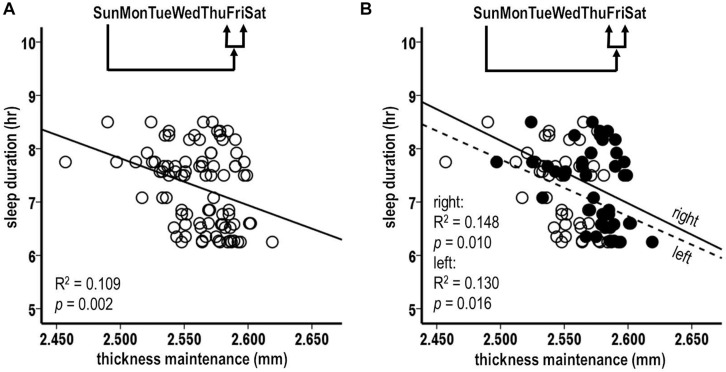
Relationships between preceding Sunday thickness maintenances and sleep durations for subsequent week Friday nights **(A)**. Laterality analyses of fluctuations in preceding Sunday thickness maintenances for separate right (filled circles, solid regression line) and left (unfilled circles, dashed regression lines) hemispheres vs. sleep durations for subsequent week Friday nights **(B)**. Preceding thickness maintenances of the hemispheres taken together **(A)** or separately **(B)** were significantly inversely related to sleep durations on subsequent Friday nights.

Pursuing analysis 6 results, relationships were assessed between preceding Sunday thickness maintenances vs. subsequent Thursday, Friday, and Saturday, i.e., following 5th, 6th, and 7th individual night sleep durations. There was a significant inverse relation between preceding thickness maintenance and the following Friday sleep durations (Figure 6A; *R*^2^ = 0.109, *p* = 0.002). In contrast, the relationships for Thursday and Saturday nights were not significant (Thursday: *R*^2^ = 0.027, *p* = 0.124; Saturday: *R*^2^ = 0.038, *p* = 0.068). *Post hoc* individual night tests for the remaining Sunday–Wednesday nights were also not significant. These results suggest a gradient in strength of preceding thickness maintenance effects on subsequent sleep durations, with stronger effects being prolonged/delayed several days.

#### Analysis 8. Laterality Analysis of Preceding Thickness Maintenances for Separate Left and Right Hemispheres vs. Sleep Duration for Subsequent Friday Nights

The above Friday finding from pooled thickness maintenances was further tested separately for the left and the right hemispheres. The preceding Sunday thickness maintenances in each hemisphere were significantly inversely related to subsequent Friday night sleep durations (Figure 6B; left: *R*^2^ = 0.130, *p* = 0.016; right: *R*^2^ = 0.148, *p* = 0.010). This suggests bilateral contributions of preceding thickness maintenance influences on subsequent sleep.

### Question 3: Do Cortical Thickness Maintenance and Sleep Duration Interact Bidirectionally Over Short Intervals?

Analyses 1–8 do not define the temporal patterns of the observed unidirectional sleep–thickness or thickness–sleep relationships nor do they test potential bidirectional interactions. This requires time series analyses that identify when the unidirectional inverse relationships in each direction occurred and whether they occurred as isolated unidirectional events over separate weeks and/or as bidirectionally interacting events across successive weeks.

This was done by testing whether higher and lower percentile mean thickness maintenances for each week were inversely related, respectively, to lower and higher percentile successively preceding and following sleep measures. This approach was used, first, for week sleep durations (Analysis 9) and, next, for individual night sleep durations (Analysis 10). Tests were done separately for each hemisphere to assess side–side effects and relationships.

#### Analysis 9. Time Series Analyses of Relationships Between Cortical Thickness Maintenance and Preceding and Subsequent Week Sleep Durations

Percentiles of mean thickness maintenances derived from the two scans on each Sunday were plotted with percentiles of mean sleep durations for Sunday–Saturday night week periods to assess sequences of inverse relationships in the sleep to thickness maintenance and thickness maintenance to sleep directions for the 44 successive week periods.

**FIGURE 7:**
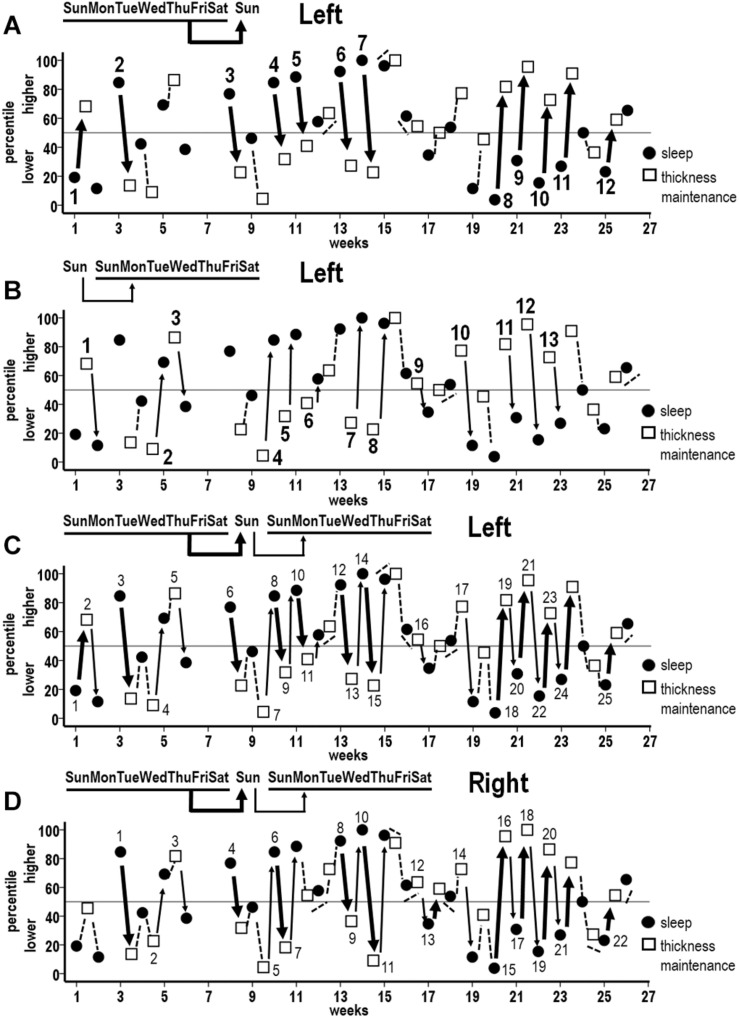
Sequences of associations between successive percentile measures for week mean sleep durations (filled circles) and thickness maintenances (unfilled squares) across the study. **(A)** Unidirectional inverse relationships between preceding week sleep duration vs. subsequent Sunday *left* hemisphere thickness maintenance: *higher* percentile preceding sleep durations vs. subsequent *lower* percentile left hemisphere thickness maintenances (broad arrows 2–7), and *lower* percentile preceding sleep durations vs. subsequent *higher* percentile left hemisphere thickness maintenances (broad arrows 1 and 8–12). **(B)** Reverse direction unidirectional inverse relationships between preceding Sunday *left* hemisphere thickness maintenance vs. subsequent week sleep duration: *higher* percentile preceding left hemisphere thickness maintenances vs. subsequent *lower* percentile sleep durations (thin arrows 1, 3, 9–13), and *lower* percentile preceding left thickness maintenances vs. subsequent *higher* percentile sleep durations (thin arrows 2, 4–8). **(C)** Combined plot of unidirectional inverse sequences from **(A,B)** to indicate sequences of unidirectional relationships involving single periods (individual broad and thin arrows 3, 4, 5, 6, 16, 17, and 25) and of bidirectional interactions across multiple successive periods (alternating broad and thin arrows 1–2, 7–11, 12–15, and 18–24) for *left* hemisphere thickness maintenance. **(D)** Combined plot indicating analogous sequences of unidirectional relationships involving single periods (individual broad and thin arrows 1, 2, 3, 4, 14, and 22) and of bidirectional interactions across multiple successive periods (alternating broad and thin arrows 5–7, 8–11, 12–13, and 15–21) for *right* hemisphere thickness maintenance. Relationships that do not fit 50th percentile inverse criteria are indicated in **(A–D)** with dashed lines.

For the left hemisphere, in the preceding sleep to subsequent thickness maintenance direction, inverse sequences were apparent where higher sleep duration percentiles for the preceding week were related to lower thickness percentiles on the subsequent Sunday (Figure 7A, broad arrows 2–7) and, conversely, where lower sleep duration percentiles for the preceding week were related to higher thickness percentiles on the subsequent Sunday (Figure 7A, broad arrows 1 and 8–12; higher and lower relative to the indicated 50th percentile). These inverse sequences were supplemented by intervening sleep-to-thickness maintenance sequences that did not fit the 50th percentile criteria (Figure 7A, dashed lines).

Analogous inverse sequences were apparent in the opposite, i.e., preceding left thickness maintenance to subsequent sleep direction. Specifically, higher thickness percentiles on the preceding Sunday were related to lower sleep duration percentiles for the subsequent week (Figure 7B, thin arrows 1, 3, and 9–13). Conversely, lower thickness percentiles on the preceding Sunday were related to higher sleep duration percentiles for the subsequent week (Figure 7B, thin arrows 2 and 4–8). These inverse sequences were supplemented by intervening thickness maintenance-to-sleep sequences that did not fit the 50th percentile criteria (Figure 7B, dashed lines).

To examine how the above unidirectional relationships (Figures 7A,B) operated with respect to each other, relationships in both directions were plotted together (Figure 7C). This indicated that inverse associations involved individual periods of unidirectional relationship in either direction (Figure 7C, broad arrows 3, 6, and 25; thin arrows 4, 5, 16, and 17), as well as continuous progressions of seesawing bidirectional interactions across two to seven successive periods (Figure 7C, alternating broad and thin arrows 1–2, 7–11, 12–15, and 18–24). Taken together, these unidirectional relationships and bidirectional interactions spanned 57% of periods across the study. These inverse sequences were supplemented by intervening sleep–thickness and thickness–sleep sequences that did not fit the 50th percentile criteria (Figure 7C, dashed lines).

Similar findings applied to the right hemisphere. A plot combining associations between preceding week sleep duration vs. subsequent right thickness maintenance and preceding right thickness maintenance vs. subsequent week sleep duration indicated inverse sequences that involved individual periods of unidirectional relationships (Figure 7D, broad arrows 1, 4, and 22; thin arrows 2, 3, and 14) as well as continuous progressions of seesawing bidirectional interactions across two to seven successive periods (Figure 7D, alternating broad and thin arrows 5–7, 8–11, 12–13, and 15–21). Taken together, these unidirectional relationships and bidirectional interactions spanned 50% of periods across the study. Sequences that did not fit the 50th percentile criteria occurred during intervening periods (Figure 7D, dashed lines).

Occurrences of inverse sequences involving week sleep durations were significantly positively related for the left and the right hemispheres (Table 1, week).

**TABLE 1:** Correlations in occurrences of the left and the right hemisphere inverse sequences.

**Sleep period**	**Correlation^*a*^**
Week	+ 0.780*
Saturday	+ 0.726*
Friday	+ 0.686*
Thursday	+ 0.812*
Wednesday	+ 0.833*
Tuesday	+ 0.815*
Monday	+ 0.821*
Sunday	+ 0.727*

*^*a*^Pearson r.* *p < 0.001.

#### Analysis 10. Time Series Analyses of Relationships Between Cortical Thickness Maintenance and Preceding and Subsequent Individual Night Sleep Durations

**FIGURE 8:**
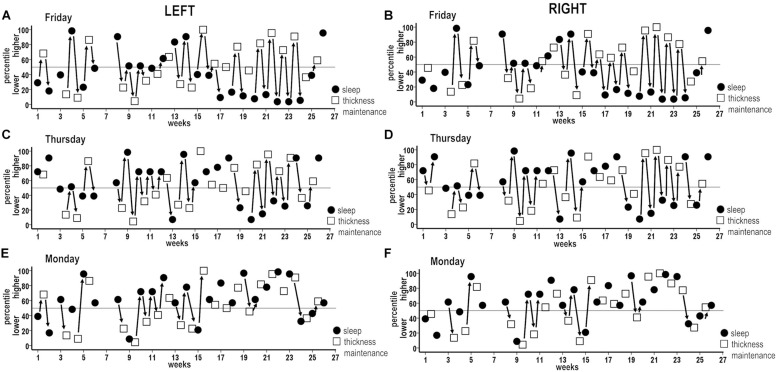
Combined plots of successive sequences of inverse associations between higher and lower and lower and higher percentile measures for individual night sleep durations (filled circles) and thickness maintenances (unfilled squares) across the study. Sequences of unidirectional relationships and bidirectional interactions are indicated (solid arrows) for respective left and right hemisphere thickness maintenances vs. Friday **(A,B)**, Thursday **(C,D)**, and Monday **(E,F)** night sleep durations. For each night, inverse sequences fitting the 50th percentile criteria were supplemented by intervening sequences that did not fit these criteria.

Analysis 9 findings for week sleep durations further applied to individual nights. Incidences of inverse associations were highest for Friday nights when the left and the right hemispheres each had inverse sequences for 68% of the successive periods across the study (Figures 8A,B, solid arrows). Analogous to Analysis 9 findings, the inverse sequences for Friday night sleep durations involved individual periods of unidirectional relationships as well as continuous progressions of seesawing bidirectional interactions across two to eight successive periods.

**FIGURE 9:**
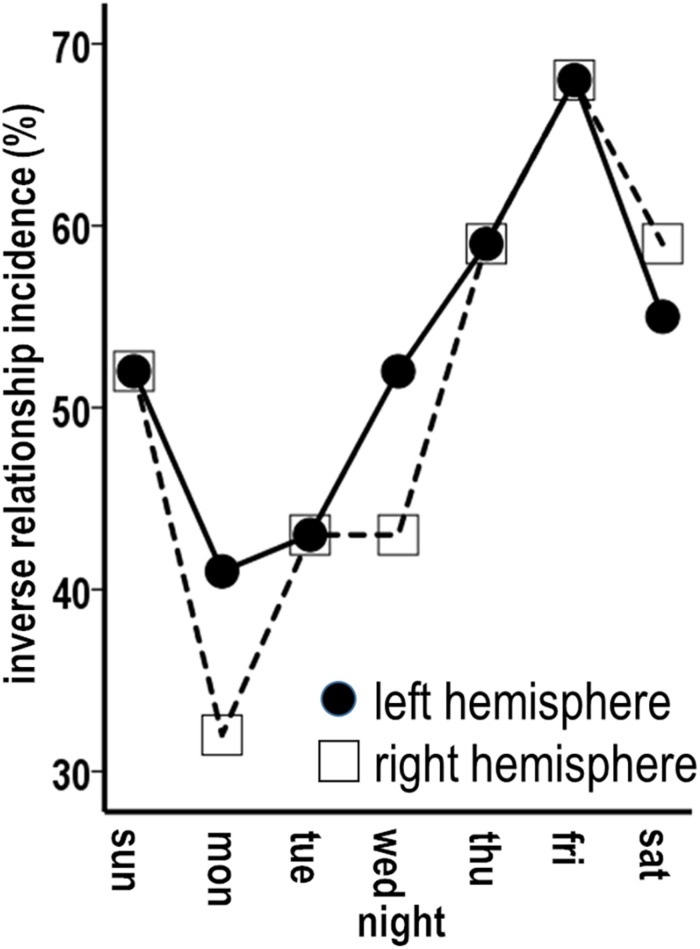
Graded incidences in occurrence of inverse associations between individual night sleep durations and left and right thickness maintenances.

These results applied to each other night to graded degrees. Higher incidences of inverse associations, also involving individual periods of unidirectional relationships as well as continuous seesawing bidirectional interactions over successive periods, occurred for Thursday nights when the left and the right hemispheres each had inverse sequences for 59% of periods (Figures 8C,D; solid arrows). Similar higher incidences (left = 55%, right = 59%) were seen for Saturday nights (Figure 9).

The remaining nights also had sequences of inverse unidirectional relationships and bidirectional interactions, but with lower incidences relative to Thursday–Saturday nights. Monday nights had left and right hemisphere inverse sequences for 41 and 32% of periods (Figures 8E,F; solid arrows). The remaining Sunday, Tuesday, and Wednesday nights had inverse sequence incidences that fell between the lower Monday and higher Thursday–Saturday night incidences (Figure 9). For all nights, inverse sequences fitting the 50th percentile criteria were supplemented by intervening sequences that did not fit these criteria (e.g., Figures 8A–F, sequences without arrows).

Stronger temporal effects in the sleep-to-thickness maintenance vs. thickness maintenance-to-sleep directions were asymmetric. For example, Thursday–Saturday nights with higher incidences of inverse sequences had prolonged/delayed effects that were shorter, i.e., involved fewer intervening nights, in the sleep–thickness maintenance, than the thickness maintenance–sleep, direction. Occurrences of inverse sequences were significantly positively related for the left and the right hemispheres for each night (Table 1).

## Discussion

### Present Findings

This is a first exploration into the interaction of sleep and cortical structural maintenance from an individual person, microlongitudinal perspective. The findings are as follow.

*Question 1: Are fluctuations in sleep duration at earlier times related to maintenance of cortical thickness at later times?* Sleep durations over nights of the preceding week had unidirectional inverse, graded, bilateral relations with subsequent thickness maintenance, with stronger effects being delayed/prolonged from the 2nd to the 3rd night before thickness measures.
*Question 2: Are fluctuations in maintenance of cortical thickness at earlier times related to sleep duration at later times?* Preceding fluctuations in cortical thickness maintenance had unidirectional inverse, graded, bilateral relations with sleep during nights of the following week, with stronger relationships being delayed/prolonged to the 5th until the 7th, especially the 6th, night after thickness measures.
*Question 3: Do cortical thickness maintenance and sleep duration interact bidirectionally over short intervals?* Time series analyses confirmed the above unidirectional relation findings and further revealed temporal dynamics and bidirectional interactions. Specifically, time series analyses revealed successive “if–then” temporal contingencies in unidirectional inverse sleep–thickness and unidirectional inverse thickness–sleep relationships which, together over successive short intervals, contributed to bidirectional inverse interactions. Inverse interactions were graded across different nights of the preceding and the following week periods, positively related across both hemispheres, and active across up to seven to eight successive weeks. Inverse interactions were also asymmetric in that stronger influences of thickness maintenance on subsequent sleep had longer delayed/prolonged effects than stronger influences of sleep on subsequent thickness maintenance.

#### Concepts From These Results

The following concepts characterize these dynamics. Inverse interactions were involved in the nature of interplay. For direction of effects, thickness maintenance and sleep uni- and bi-directionally inversely interacted in “if–then” ways. Time periods of inverse interactions involved preceding and following week periods, with graded effects across the nights of these periods. There was asymmetry in effects, with stronger inverse effects involving shorter intervals in the sleep-to-thickness maintenance, than thickness maintenance-to-sleep, direction. For laterality, the inverse effects across the two hemispheres were positively related. Finally, inverse interactions could occur continuously over successive days–weeks. To our knowledge, there have been no other attempts to define concepts of ongoing continuous interplay between sleep and cortical structural maintenance “-omics” at an individual level.

#### Generalizability of Findings

It is unlikely that the present investigation serendipitously studied the only individual to whom these concepts apply. Thus, on one hand, they arguably generalize to other individuals to some presently unknown extent. In addition, however, individuals clearly differ and are individual specific in terms of thickness and other properties of cortical structure (Mueller et al., 2013; Zilles and Amunts, 2013; Wachinger et al., 2015; Henssen et al., 2016; de Manzano and Ullen, 2018; Kruggel, 2018; Valizadeh et al., 2018; Doucet et al., 2019) and in terms of duration and other aspects of sleep (Van Dongen et al., 2005; Lewandowski et al., 2013; Saletin et al., 2013; Rusterholz et al., 2017; Chaput et al., 2018). This suggests that different expressions of the above concepts and/or different concepts apply in other individuals. These views point to the need to explore the generalizability issue with further microlongitudinal *n*-of-1 analyses.

### Do the Findings Reflect Normal Interactions in This Individual?

The following points suggest that the findings reflect normal interactions in this individual.

First, the medical history, brain scans, daily health measures, and related physician reviews indicated that the individual was in normal health. Cerebral blood flow velocities were normal and allostatic load was low. Daily survey responses indicated that he felt happy, mentally alert, and healthy and did not suffer from anxiety or depression.

**FIGURE 10:**
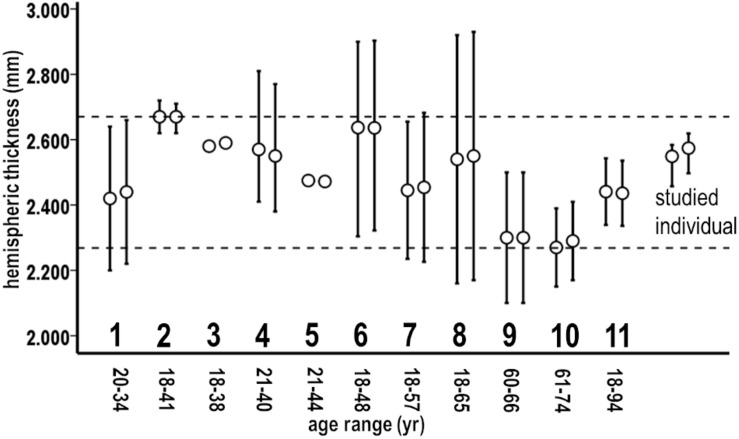
Means and variations of respective left and right cortical hemispheric thicknesses as measured with FreeSurfer in recent studies of 11 normal adult groups (1–11) having indicated age ranges, compared to the studied individual. Variation bars reflect 95% confidence intervals from reported standard deviations (1, 2, 7, 8, 9, and 10), ranges (4 and 6), or root mean square errors (11). Group 3 variations were not reported, and group 5 variations were very small (*SD* = 0.002). Dashed lines bracket the upper and lower range of means of these groups. Means of thickness maintenances in the studied individual fell within this range. From: group 1 (Tremblay and Deschamps, 2016), 2 (McGuire et al., 2017), 3 (Kang et al., 2012), 4 (Ottino-Gonzalez et al., 2017), 5 (van Erp et al., 2018), 6 (Meyer et al., 2014), 7 (Maingault et al., 2016), 8 (Perlman et al., 2017), 9 (Shaw et al., 2016), 10 (Tremblay and Deschamps, 2016), and 11 (Potvin et al., 2017).

Second, the thickness maintenances of each hemisphere were consistent with normal thicknesses of healthy adults. More specifically, his thickness maintenance means and ranges were encompassed within mean measures of hemispheric thicknesses from studies that applied FreeSurfer thickness measurement procedures to normal adult groups with age ranges that spanned or were less than his age (Figure 10).

Finally, the individual did not suffer from sleep disorders and maintained his pre-study healthy sleep habits during the study. Daily survey responses indicated that he felt rested upon awakening, had high energy, and did not experience changes in sleep, difficulty concentrating, or tiredness/fatigue. His sleep durations were regular and fluctuated within adult population guidelines for normal sleep. For example, national advisory groups have suggested 7–9 h/night (NIH, 2011; Consensus Conference et al., 2015; CDC, 2019), the National Sleep Foundation further considers as may-be-appropriate 5–7 h (Hirshkowitz et al., 2015a, b), and a recent National Health Interview Survey considered ≥ 6 h as adequate (Sheehan et al., 2019). Compared to these guidelines, his sleep reversibly fluctuated around a mean duration of 7.6 h/night and, for 98% of nights, remained within guideline ranges of > 6 to < 9 h/night.

These views suggest that the individual was in normal health and had thickness maintenance and sleep duration fluctuations that were within normal adult population ranges and guidelines. This suggests that the present findings reflect normal interaction dynamics in this individual.

### What Mechanisms Mediate Normal Interplay Between Sleep and Thickness Maintenance in This Individual?

The fluctuations in the studied individual’s thickness maintenance were previously proposed to result from continuously ongoing, normal remodeling/turnover in cortical substrates (Wall et al., 2017). These substrates are neurons and glia with associated processes and neuropil, arterial–capillary–venous vasculature cells, and intracellular/extracellular/vascular fluid and related spaces (Bennett, 2011; Kays et al., 2012; Thomas et al., 2012). Studies in normal adult animals suggest that all these substrates normally fluctuate over hours, days, and weeks. As previously reviewed in detail (Wall et al., 2017), ongoing fluctuations include, e.g., extensions and retractions of axonal branches and boutons, dendrites and spines, and glial processes. Further fluctuations involve ongoing cell loss, angiogenesis, gliogenesis, and fluxes in volumes of cells and intracellular, extracellular, and vascular fluid spaces. The involved short time periods, distances of spatial change, and high substrate densities arguably suggest that these normal substrate fluctuations result in continuous remodeling/turnover that becomes further expressed by normal fluctuation in ongoing thickness maintenance (Wall et al., 2017).

From the present findings, we propose that his normal sleep–wake cycle influenced, and was influenced by, remodeling/turnover of cortical substrates *via* interplay with normal circadian/allostatic/homeostatic mechanisms. This proposal gets support from animal and human work.

For example, animal work suggests that sleep normally interacts with combined circadian/allostatic/homeostatic mechanisms that affect cortical structure *via* regulating influences on, e.g., (a) cell maintenance — transcription, translation, metabolism, mitochondrial function, antioxidant response, and synthesis of neurochemicals (Zelinski et al., 2014; Logan and McClung, 2019), (b) astrocyte surface/volume ratio and extents of appositions with neurons (Bellesi et al., 2015), (c) length and arborization of dendrites (Karatsoreos et al., 2011; Havekes et al., 2016), and (d) number, volume, and morphology of synapses (Raven et al., 2018). A recent review of the synaptic homeostasis view suggests that trillions of synapses undergo slimming by ≈20% over a night of sleep (Cirelli and Tononi, 2017).

Sleep–wake cycle-related circadian/allostatic/homeostatic mechanisms also affect cortical substrates *via* glymphatic functions. For example, nightly sleep activation of the glymphatic system is normally accompanied by a 60% increase in cortical interstitial space volume and related increases in cerebrospinal fluid (CSF) flow, interstitial fluid, delivery of nutrients, and toxic metabolic waste clearance (Xie et al., 2013; Jessen et al., 2015; Boespflug and Iliff, 2018).

Supplementing animal work, human group studies have begun to document normal sleep–wake cycle effects on cortical thickness, fluid movement near cortical edges, and sleep delta wave coupled hemodynamic and CSF flow rhythms (Hodkinson et al., 2014; Trefler et al., 2016; Elvsashagen et al., 2017, 2019; Thomas et al., 2018; Fultz et al., 2019). These effects also involve mediation by circadian/allostatic/homeostatic mechanisms.

From these lines of evidence, we propose that the observed bidirectional interplay involved normal fluctuations in sleep that interacted with circadian/allostatic/homeostatic mechanisms that influenced subsequent normal dynamics of cortical substrate remodeling/turnover and thickness maintenance. This, in turn, contributed to cortical interactions with circadian/allostatic/homeostatic mechanisms to influence subsequent normal sleep dynamics.

### What Mechanisms May Relate Specifically to the Observed Inverse Dynamics in This Individual?

We suggest that, in the sleep-to-thickness direction, the studied individual’s normal sleep fluctuations interacted with correspondingly varying circadian/allostatic/homeostatic mechanisms which affected subsequent normal cortical substrate remodeling/turnover. For example, as indicated above, preceding shorter durations of normal sleep might have translated into shorter/lower sleep-related cortical clearance of CSF, interstitial fluid, and metabolic waste and/or less sleep-related downscaling of synaptic neuropil substrates. These effects could arguably contribute to normal substrate remodeling/turnover, leading to inverse higher normal thickness maintenance levels. With longer normal preceding sleep durations, these effects may have been reversed and contributed to inverse lower normal thickness maintenance levels.

In the thickness-to-sleep direction, his fluctuations over higher and lower normal thickness maintenance levels may have correspondingly variably interacted with circadian/allostatic/homeostatic mechanisms to affect subsequent normal sleep. For example, preceding higher normal thickness maintenance levels may have been associated with the above effects which, in turn, may have contributed to subsequent inversely related shorter normal duration sleep. Alternatively, with preceding lower normal thickness maintenance levels, these effects may have been reversed to contribute to subsequent inversely related longer normal-duration sleep.

These possibilities could be further examined by extending the scope of the present study, which addressed potential temporal relationships between spontaneously ongoing normal fluctuations in sleep duration and thickness maintenance, to a study where the subject deliberately regulates his sleep durations in downward and upward directions to the limits of normal sleep guidelines. This could serve as a further test for inverse interactions in the sleep–thickness maintenance and thickness maintenance–sleep directions.

The above views share consistencies with glymphatic and synaptic homeostasis viewpoints on sleep and its normal effects on cortical substrates (Lucey and Holtzman, 2015; Nedergaard and Goldman, 2016; Cirelli and Tononi, 2017; de Vivo et al., 2017; Diering et al., 2017; Boespflug and Iliff, 2018; Rasmussen et al., 2018; Shokri-Kojori et al., 2018).

### Implications

The present findings have three implications.

Implication 1: an individual’s cortical thickness maintenance and sleep can, under normal conditions, be bidirectionally related.

Existing sleep and cortical MRI work leads to an impasse in defining the direction(s) of normal interplay between sleep and cortical thickness largely because it focuses on group average observations from one or few sleep and thickness measures per person and/or on between-measure times that are too long or irregular, for identifying the direction(s) of relationships. The present individual-focused, microlongitudinal time series analyses suggest that normal sleep duration and normal thickness maintenance interacted bidirectionally. It is difficult to completely rule out the possibility that some spurious factor(s) explain the present results. However, the findings that directional contingencies occurred repeatedly, at short intervals, with graded impacts for specific nights, over successive weeks and bilaterally, point to patterns that are consistent with temporal “if–then” contingencies between sleep and thickness maintenance. This claim merits attention and points to a need for further microlongitudinal *n*-of-1 studies.

Implication 2: interaction dynamics between normal cortical thickness maintenance and sleep in the studied individual appear to involve or even require interactions with other factors.

This implication arises from the following considerations. First, the regression lines for significant inverse sleep–thickness and thickness–sleep relationships had *R*^2^-values of, e.g., 0.059 for week and 0.190 for individual night sleep periods. In addition, the time sequence analyses indicated that 32–68% of periods for different individual nights had inverse relationships. These results leave room for influences of other factors.

Second, sleep and cortical structure are each known to influence and be influenced by a range of factors. For example, each is integrated within dynamic, non-linear, reciprocally interactive circadian/allostatic/homeostatic mechanisms which interact with cognitive, autonomic, genetic, immunological, hormonal, metabolic, and other factors (Wulff et al., 2010; Zelinski et al., 2014; Carroll et al., 2015; McEwen and Karatsoreos, 2015; Musiek and Holtzman, 2016; Logan and McClung, 2019). The present correlation and time sequence results clearly leave room for substantial influences of these or other factors that were not measured. A comprehensive understanding of multi-factor interaction dynamics between sleep and cortical structural “-omics” in an individual is not yet available but is a viable future target for precision health maintenance research.

Implication 3: microlongitudinal n-of-1 analysis may prove useful for understanding how to optimize the interaction of cortical structural maintenance and sleep in the studied individual.

Paralleling the limited understanding of what constitutes optimal sleep (Blunden and Galland, 2014; Matricciani et al., 2017; Chaput et al., 2018), concepts that define an individual person’s optimal sleep duration, brain maintenance, and related interactions also remain unclear.

The present findings may be helpful for promoting cortical health and avoiding thickness maintenance problems in the studied individual. For example, as discussed above, nights with normal shorter sleep durations may result in lower normal cortical interstitial fluid and metabolic waste clearance and reduced neuropil downscaling which, in turn, could subsequently contribute to higher normal thickness maintenance levels. These higher levels appeared reversible with intervening nights of normal longer sleep durations. However, this same higher thickness maintenance, if persistent due to repeated short or disrupted sleep, may result in prodromal detrimental metabolic or pro-inflammatory effects on substrate remodeling/turnover that may promote eventual thickness thinning as commonly occurs with comorbid sleep disorders and cortical degenerative diseases (Ju et al., 2014; Sanchez-Espinosa et al., 2014; Suh et al., 2016; Cedernaes et al., 2017; Macey et al., 2018). Potentially also contributing, consistently short nightly sleep durations may result in shorter or fewer periods of healthy sleep-related cortical activity and disruption of normal hemodynamic and CSF rhythms (Fultz et al., 2019; Hablitz et al., 2019). This may also adversely impact cortical substrate remodeling/turnover and thickness maintenance. Thus, a shift in this individual’s sleep habits from consistently reversing shorter and longer normal sleep durations to, instead, consistently short sleep durations may lead to different consequences for his thickness maintenance.

As can be seen in the present study, a microlongitudinal *n*-of-1 time series approach can begin to provide data on personal patterns of healthy sleep and cortical maintenance interactions over day–week–month times. This raises the provocative possibility of using an individual’s idiosyncratic patterns of sleep and thickness maintenance “-omics” to proactively identify early preclinical dispositions for cortical degenerative and related mental/cognitive disorders. This could contribute to filling acknowledged gaps in understanding of sleep and brain health (Fung et al., 2016).

### Limitations

This study has clear limitations: (1) the data are from one person and were collected to assess concepts in this person, (2) cortical structural maintenance is assessed only in terms of thickness, (3) sleep is assessed in terms of duration, not with actigraphy, polysomnography, or other sleep dimensions, and (4) it is meant as a starting, not finishing, line for understanding the addressed questions at an individual-person level.

## Conclusion

There currently is no conceptualization of how normal structural maintenance of an individual person’s brain interacts with sleep on a day–day and week–week basis. The present microlongitudinal time series analyses reveal normal dynamics of uni- and bi-directional interactions between maintenance of cortical structure and sleep that are currently not recognized at an individual-person level. These interactions are suggested to result from cortical and sleep influences on normal circadian/allostatic/homeostatic mechanisms that affect sleep and remodeling/turnover of cortical substrates. These *n*-of-1 findings have implications for precision medicine use of microlongitudinal time series data from a particular person to optimize that person’s sleep and brain health.

## Data Availability Statement

Raw data generated in this study are not publicly available to protect ethical, legal, and privacy concerns of the studied individual. Address questions to JW at john.wall@utoledo.edu.

## Ethics Statement

This study was reviewed and approved by the University of Toledo Institutional Review Board and done with the written informed consent of the subject. Written informed consent was obtained from the individual for the publication of any potentially identifiable images or data included in this article.

## Author Contributions

JW, HX, and XW conceived and designed the experiment. JW and XW contributed to data acquisition. JW and HX analyzed the data, wrote the manuscript, and developed the structure and arguments for the manuscript. All authors reviewed and approved the final version.

## Conflict of Interest

The authors declare that the research was conducted in the absence of any commercial or financial relationships that could be construed as a potential conflict of interest.

## Footnotes

1 http://surfer.nmr.mgh.harvard.edu

## References

[B1] Alberti K. G. Eckel R. H. Grundy S. M. Zimmet P. Z. Cleeman J. I. Donato K. A. (2009). Harmonizing the metabolic syndrome: a joint interim statement of the International Diabetes Federation Task Force on Epidemiology and Prevention; National Heart, Lung, and Blood Institute; American Heart Association; World Heart Federation; International Atherosclerosis Society; and International Association for the Study of Obesity. *Circulation* 120 1640–1645. 10.1161/circulationaha.109.192644 19805654

[B2] Barth C. Steele C. J. Mueller K. Rekkas V. P. Arélin K. Pampel A. (2016). In-vivo dynamics of the human hippocampus across the menstrual cycle. *Sci. Rep.* 6:32833. 10.1038/srep32833 27713470 PMC5054394

[B3] Bellesi M. de Vivo L. Tononi G. Cirelli C. (2015). Effects of sleep and wake on astrocytes: clues from molecular and ultrastructural studies. *BMC Biol.* 13:66. 10.1186/s12915-015-0176-7 26303010 PMC4548305

[B4] Bennett M. R. (2011). The prefrontal-limbic network in depression: a core pathology of synapse regression. *Prog. Neurobiol.* 93 457–467. 10.1016/j.pneurobio.2011.01.001 21335052

[B5] Blunden S. Galland B. (2014). The complexities of defining optimal sleep: empirical and theoretical considerations with a special emphasis on children. *Sleep Med. Rev.* 18 371–378. 10.1016/j.smrv.2014.01.002 24629828

[B6] Boespflug E. L. Iliff J. J. (2018). The emerging relationship between interstitial fluid-cerebrospinal fluid exchange, amyloid-beta, and sleep. *Biol. Psychiatry* 83 328–336. 10.1016/j.biopsych.2017.11.031 29279202 PMC5767516

[B7] Braga R. M. Buckner R. L. (2017). Parallel interdigitated distributed networks within the individual estimated by intrinsic functional connectivity. *Neuron* 95 457–471.e5. 10.1016/j.neuron.2017.06.038 28728026 PMC5519493

[B8] Carroll J. E. Irwin M. R. Stein Merkin S. Seeman T. E. (2015). Sleep and multisystem biological risk: a population-based study. *PLoS One* 10:e0118467. 10.1371/journal.pone.0118467 25714703 PMC4340787

[B9] CDC (2019). *How Much Sleep do I Need? Centers for Disease Control and Prevention website.* Available online at: https://www.cdc.gov/features/sleep/index.html (accessed July 21, 2020).

[B10] Cedernaes J. Osorio R. S. Varga A. W. Kam K. Schioth H. B. Benedict C. (2017). Candidate mechanisms underlying the association between sleep-wake disruptions and Alzheimer’s disease. *Sleep Med. Rev.* 31 102–111. 10.1016/j.smrv.2016.02.002 26996255 PMC4981560

[B11] Chaput J.-P. Dutil C. Sampasa-Kanyinga H. (2018). Sleeping hours: what is the ideal number and how does age impact this? *Nat. Sci. Sleep* 10 421–430. 10.2147/NSS.S163071 30568521 PMC6267703

[B12] Chen R. Xia L. Tu K. Duan M. Kukurba K. Li-Pook-Than J. (2018). Longitudinal personal DNA methylome dynamics in a human with a chronic condition. *Nat. Med.* 24 1930–1939. 10.1038/s41591-018-0237-x 30397358 PMC8084418

[B13] Chen X. Redline S. Shields A. E. Williams D. R. Williams M. A. (2014). Associations of allostatic load with sleep apnea, insomnia, short sleep duration, and other sleep disturbances: findings from the National Health and Nutrition Examination Survey 2005 to 2008. *Ann. Epidemiol.* 24 612–619. 10.1016/j.annepidem.2014.05.014 24985316 PMC4188508

[B14] Choe A. S. Jones C. K. Joel S. E. Muschelli J. Belegu V. Caffo B. S. (2015). Reproducibility and temporal structure in weekly resting-state fMRI over a period of 3.5 years. *PLoS One* 10:e0140134. 10.1371/journal.pone.0140134 26517540 PMC4627782

[B15] Cirelli C. Tononi G. (2017). The sleeping brain. *Cerebrum* 2017:cer-07-17. PMC5501041 28698776

[B16] Cole J. H. (2018). Neuroimaging studies illustrate the commonalities between ageing and brain diseases. *Bioessays* 40:e1700221. 10.1002/bies.201700221 29882974

[B17] Collins F. S. Varmus H. (2015). A new initiative on precision medicine. *N. Engl. J. Med.* 372 793–795. 10.1056/NEJMp1500523 25635347 PMC5101938

[B18] Consensus Conference P. Watson N. F. Badr M. S. Belenky G. Bliwise D. L. Buxton O. M. (2015). Recommended amount of sleep for a healthy adult: a joint consensus statement of the American Academy of Sleep Medicine and Sleep Research Society. *J. Clin. Sleep Med.* 11 591–592. 10.5664/jcsm.4758 25979105 PMC4442216

[B19] David L. A. Materna A. C. Friedman J. Campos-Baptista M. I. Blackburn M. C. Perrotta A. (2014). Host lifestyle affects human microbiota on daily timescales. *Genome Biol.* 15:R89. 10.1186/gb-2014-15-7-r89 25146375 PMC4405912

[B20] de Manzano O. Ullen F. (2018). Same genes, different brains: neuroanatomical differences between monozygotic twins discordant for musical training. *Cereb. Cortex* 28 387–394. 10.1093/cercor/bhx299 29136105

[B21] de Vivo L. Bellesi M. Marshall W. Bushong E. A. Ellisman M. H. Tononi G. (2017). Ultrastructural evidence for synaptic scaling across the wake/sleep cycle. *Science* 355 507–510. 10.1126/science.aah5982 28154076 PMC5313037

[B22] Diering G. H. Nirujogi R. S. Roth R. H. Worley P. F. Pandey A. Huganir R. L. (2017). Homer1a drives homeostatic scaling-down of excitatory synapses during sleep. *Science* 355 511–515. 10.1126/science.aai8355 28154077 PMC5382711

[B23] Donnelly-Kehoe P. Saenger V. M. Lisofsky N. Kuhn S. Kringelbach M. L. Schwarzbach J. (2019). Reliable local dynamics in the brain across sessions are revealed by whole-brain modeling of resting state activity. *Hum. Brain Mapp.* 40 2967–2980. 10.1002/hbm.24572 30882961 PMC6865451

[B24] Doucet G. E. Moser D. A. Rodrigue A. Bassett D. S. Glahn D. C. Frangou S. (2019). Person-based brain morphometric similarity is heritable and correlates with biological features. *Cereb. Cortex* 29 852–862. 10.1093/cercor/bhy287 30462205 PMC6319174

[B25] Elvsashagen T. Mutsaerts H. J. Zak N. Norbom L. B. Quraishi S. H. Pedersen P. O. (2019). Cerebral blood flow changes after a day of wake, sleep, and sleep deprivation. *Neuroimage* 186 497–509. 10.1016/j.neuroimage.2018.11.032 30471387

[B26] Elvsashagen T. Zak N. Norbom L. B. Pedersen P. O. Quraishi S. H. Bjornerud A. (2017). Evidence for cortical structural plasticity in humans after a day of waking and sleep deprivation. *Neuroimage* 156 214–223. 10.1016/j.neuroimage.2017.05.027 28526620

[B27] Filevich E. Lisofsky N. Becker M. Butler O. Lochstet M. Mårtensson J. (2017). Day2day: Investigating daily variability of magnetic resonance imaging measures over half a year. *BMC Neurosci.* 18:65. 10.1186/s12868-017-0383-y 28836958 PMC5571657

[B28] Fischer D. Lombardi D. A. Marucci-Wellman H. Roenneberg T. (2017). Chronotypes in the US - Influence of age and sex. *PLoS One* 12:e0178782. 10.1371/journal.pone.0178782 28636610 PMC5479630

[B29] Fischl B. (2012). FreeSurfer. *Neuroimage* 62 774–781. 10.1016/j.neuroimage.2012.01.021 22248573 PMC3685476

[B30] Fultz N. E. Bonmassar G. Setsompop K. Stickgold R. A. Rosen B. R. Polimeni J. R. (2019). Coupled electrophysiological, hemodynamic, and cerebrospinal fluid oscillations in human sleep. *Science* 366 628–631. 10.1126/science.aax5440 31672896 PMC7309589

[B31] Fung C. H. Vitiello M. V. Alessi C. A. Kuchel G. A. (2016). Report and Research Agenda of the American Geriatrics Society and National Institute on Aging Bedside-to-Bench Conference on Sleep, Circadian Rhythms, and Aging: new Avenues for Improving Brain Health, Physical Health, and Functioning. *J. Am. Geriat. Soc.* 64 e238–e247. 10.1111/jgs.14493 27858974 PMC5173456

[B32] Ganzel B. L. Morris P. A. Wethington E. (2010). Allostasis and the human brain: integrating models of stress from the social and life sciences. *Psychol. Rev.* 117 134–174. 10.1037/a0017773 20063966 PMC2808193

[B33] Goetz L. H. Schork N. J. (2018). Personalized medicine: motivation, challenges, and progress. *Fertil. Steril.* 109 952–963. 10.1016/j.fertnstert.2018.05.006 29935653 PMC6366451

[B34] Gordon E. M. Laumann T. O. Gilmore A. W. Newbold D. J. Greene D. J. Berg J. J. (2017). Precision functional mapping of individual human brains. *Neuron* 95 791–807.e7. 10.1016/j.neuron.2017.07.011 28757305 PMC5576360

[B35] Hablitz L. M. Vinitsky H. S. Sun Q. Stæger F. F. Sigurdsson B. Mortensen K. N. (2019). Increased glymphatic influx is correlated with high EEG delta power and low heart rate in mice under anesthesia. *Sci. Adv.* 5:eaav5447. 10.1126/sciadv.aav5447 30820460 PMC6392807

[B36] Havekes R. Park A. J. Tudor J. C. Luczak V. G. Hansen R. T. Ferri S. L. (2016). Sleep deprivation causes memory deficits by negatively impacting neuronal connectivity in hippocampal area CA1. *eLife* 5:e13424. 10.7554/eLife.13424 27549340 PMC4996653

[B37] Henssen A. Zilles K. Palomero-Gallagher N. Schleicher A. Mohlberg H. Gerboga F. (2016). Cytoarchitecture and probability maps of the human medial orbitofrontal cortex. *Cortex* 75 87–112. 10.1016/j.cortex.2015.11.006 26735709

[B38] Hirshkowitz M. Whiton K. Albert S. M. Alessi C. Bruni O. DonCarlos L. (2015a). National Sleep Foundation’s updated sleep duration recommendations: final report. *Sleep Health* 1 233–243. 10.1016/j.sleh.2015.10.004 29073398

[B39] Hirshkowitz M. Whiton K. Albert S. M. Alessi C. Bruni O. DonCarlos L. (2015b). National Sleep Foundation’s sleep time duration recommendations: methodology and results summary. *Sleep Health* 1 40–43. 10.1016/j.sleh.2014.12.010 29073412

[B40] Hodkinson D. J. O’Daly O. Zunszain P. A. Pariante C. M. Lazurenko V. Zelaya F. O. (2014). Circadian and homeostatic modulation of functional connectivity and regional cerebral blood flow in humans under normal entrained conditions. *J. Cereb. Blood Flow Metab.* 34 1493–1499. 10.1038/jcbfm.2014.109 24938404 PMC4158665

[B41] Huang S. Hood L. (2019). Personalized, precision, and N-of-one medicine: a clarification of terminology and concepts. *Perspect. Biol. Med.* 62 617–639. 10.1353/pbm.2019.0036 31761797

[B42] Insel T. R. Cuthbert B. N. (2015). Medicine. Brain disorders? Precisely. *Science* 348 499–500. 10.1126/science.aab2358 25931539

[B43] Jessen N. A. Munk A. S. Lundgaard I. Nedergaard M. (2015). The glymphatic system: a beginner’s guide. *Neurochem. Res.* 40 2583–2599. 10.1007/s11064-015-1581-6 25947369 PMC4636982

[B44] Jiang C. Wang X. Li X. Inlora J. Wang T. Liu Q. (2018). Dynamic human environmental exposome revealed by longitudinal personal monitoring. *Cell* 175 277–291.e31. 10.1016/j.cell.2018.08.060 30241608 PMC6472932

[B45] Jones S. E. Lane J. M. Wood A. R. van Hees V. T. Tyrrell J. Beaumont R. N. (2019). Genome-wide association analyses of chronotype in 697,828 individuals provides insights into circadian rhythms. *Nat. Commun.* 10:343. 10.1038/s41467-018-08259-7 30696823 PMC6351539

[B46] Ju Y.-E. S. Lucey B. P. Holtzman D. M. (2014). Sleep and Alzheimer disease pathology–a bidirectional relationship. *Nat. Rev. Neurol.* 10 115–119. 10.1038/nrneurol.2013.269 24366271 PMC3979317

[B47] Juster R. P. McEwen B. S. Lupien S. J. (2010). Allostatic load biomarkers of chronic stress and impact on health and cognition. *Neurosci. Biobehav. Rev.* 35 2–16. 10.1016/j.neubiorev.2009.10.002 19822172

[B48] Kang X. Herron T. J. Cate A. D. Yund E. W. Woods D. L. (2012). Hemispherically-unified surface maps of human cerebral cortex: reliability and hemispheric asymmetries. *PLoS One* 7:e45582. 10.1371/journal.pone.0045582 23029115 PMC3445499

[B49] Karatsoreos I. N. Bhagat S. Bloss E. B. Morrison J. H. McEwen B. S. (2011). Disruption of circadian clocks has ramifications for metabolism, brain, and behavior. *Proc. Natl. Acad. Sci. U.S.A.* 108 1657–1662. 10.1073/pnas.1018375108 21220317 PMC3029753

[B50] Kays J. L. Hurley R. A. Taber K. H. (2012). The dynamic brain: neuroplasticity and mental health. *J. Neuropsychiatry Clin. Neurosci.* 24 118–124. 10.1176/appi.neuropsych.12050109 22772660

[B51] Kruggel F. (2018). The macro-structural variability of the human neocortex. *Neuroimage* 172 620–630. 10.1016/j.neuroimage.2018.01.074 29410357

[B52] Laumann T. O. Gordon E. M. Adeyemo B. Snyder A. Z. Joo S. J. Chen M. Y. (2015). Functional system and areal organization of a highly sampled individual human brain. *Neuron* 87 657–670. 10.1016/j.neuron.2015.06.037 26212711 PMC4642864

[B53] Lewandowski A. Rosipal R. Dorffner G. (2013). On the individuality of sleep EEG spectra. *J. Psychophysiol.* 27 105–112. 10.1027/0269-8803/a000092 23997385 PMC3755818

[B54] Li X. Dunn J. Salins D. Zhou G. Zhou W. Schüssler-Fiorenza Rose S. M. (2017). Digital health: tracking physiomes and activity using wearable biosensors reveals useful health-related information. *PLoS Biol.* 15:e2001402. 10.1371/journal.pbio.2001402 28081144 PMC5230763

[B55] Logan R. W. McClung C. A. (2019). Rhythms of life: circadian disruption and brain disorders across the lifespan. *Nat. Rev. Neurosci.* 20 49–65. 10.1038/s41583-018-0088-y 30459365 PMC6338075

[B56] Lucey B. P. Holtzman D. M. (2015). How amyloid, sleep and memory connect. *Nat. Neurosci.* 18 933–934. 10.1038/nn.4048 26108720 PMC4770804

[B57] Macey P. M. Haris N. Kumar R. Thomas M. A. Woo M. A. Harper R. M. (2018). Obstructive sleep apnea and cortical thickness in females and males. *PLoS One* 13:e0193854. 10.1371/journal.pone.0193854 29509806 PMC5839576

[B58] Maclaren J. Han Z. Vos S. B. Fischbein N. Bammer R. (2014). Reliability of brain volume measurements: a test-retest dataset. *Sci. Data* 1:140037. 10.1038/sdata.2014.37 25977792 PMC4411010

[B59] Maingault S. Tzourio-Mazoyer N. Mazoyer B. Crivello F. (2016). Regional correlations between cortical thickness and surface area asymmetries: a surface-based morphometry study of 250 adults. *Neuropsychologia* 93 350–364. 10.1016/j.neuropsychologia.2016.03.025 27020136

[B60] Matricciani L. Bin Y. S. Lallukka T. Kronholm E. Dumuid D. Paquet C. (2017). Past, present, and future: trends in sleep duration and implications for public health. *Sleep Health* 3 317–323. 10.1016/j.sleh.2017.07.006 28923186

[B61] McEwen B. S. Getz L. (2013). Lifetime experiences, the brain and personalized medicine: an integrative perspective. *Metabolism* 62 (Suppl. 1), S20–S26. 10.1016/j.metabol.2012.08.020 23009787

[B62] McEwen B. S. Karatsoreos I. N. (2015). Sleep deprivation and circadian disruption: stress, allostasis, and allostatic load. *Sleep Med. Clin.* 10 1–10. 10.1016/j.jsmc.2014.11.007 26055668 PMC8935364

[B63] McGuire S. A. Wijtenburg S. A. Sherman P. M. Rowland L. M. Ryan M. Sladky J. H. (2017). Reproducibility of quantitative structural and physiological MRI measurements. *Brain Behav.* 7:e00759. 10.1002/brb3.759 28948069 PMC5607538

[B64] Meyer M. Liem F. Hirsiger S. Jancke L. Hanggi J. (2014). Cortical surface area and cortical thickness demonstrate differential structural asymmetry in auditory-related areas of the human cortex. *Cereb. Cortex* 24 2541–2552. 10.1093/cercor/bht094 23645712

[B65] Mueller S. Wang D. Fox M. D. Yeo B. T. Sepulcre J. Sabuncu M. R. (2013). Individual variability in functional connectivity architecture of the human brain. *Neuron* 77 586–595. 10.1016/j.neuron.2012.12.028 23395382 PMC3746075

[B66] Musiek E. S. Holtzman D. M. (2016). Mechanisms linking circadian clocks, sleep, and neurodegeneration. *Science* 354 1004–1008. 10.1126/science.aah4968 27885006 PMC5219881

[B67] Nedergaard M. Goldman S. A. (2016). Brain drain. *Sci. Am.* 314 44–49. 10.1038/scientificamerican0316-44 PMC5347443 27066643

[B68] NIH (2011). *Your Guide to Healthy Sleep. National Institutes of Health Publication No. 11-5271.* Avaialble at: https://www.nhlbi.nih.gov/files/docs/public/sleep/healthy_sleep.pdf (accessed July 21, 2020).

[B69] Ottino-Gonzalez J. Jurado M. A. Garcia-Garcia I. Segura B. Marques-Iturria I. Sender-Palacios M. J. (2017). Allostatic load is linked to cortical thickness changes depending on body-weight status. *Front. Hum. Neurosci.* 11:639. 10.3389/fnhum.2017.00639 29375342 PMC5770747

[B70] Parente V. Hale L. Palermo T. (2013). Association between breast cancer and allostatic load by race: national Health and Nutrition Examination Survey 1999-2008. *Psychooncology* 22 621–628. 10.1002/pon.3044 22290849

[B71] Perlman G. Bartlett E. DeLorenzo C. Weissman M. McGrath P. Ogden T. (2017). Cortical thickness is not associated with current depression in a clinical treatment study. *Hum. Brain Mapp.* 38 4370–4385. 10.1002/hbm.23664 28594150 PMC5546998

[B72] Poldrack R. A. Laumann T. O. Koyejo O. Gregory B. Hover A. Chen M. Y. (2015). Long-term neural and physiological phenotyping of a single human. *Nat. Commun.* 6:8885. 10.1038/ncomms9885 26648521 PMC4682164

[B73] Potvin O. Dieumegarde L. Duchesne S. (2017). Normative morphometric data for cerebral cortical areas over the lifetime of the adult human brain. *Neuroimage* 156 315–339. 10.1016/j.neuroimage.2017.05.019 28512057

[B74] Rasmussen M. K. Mestre H. Nedergaard M. (2018). The glymphatic pathway in neurological disorders. *Lancet Neurol.* 17 1016–1024. 10.1016/s1474-4422(18)30318-1 30353860 PMC6261373

[B75] Raven F. Van der Zee E. A. Meerlo P. Havekes R. (2018). The role of sleep in regulating structural plasticity and synaptic strength: implications for memory and cognitive function. *Sleep Med. Rev.* 39 3–11. 10.1016/j.smrv.2017.05.002 28641933

[B76] Rusterholz T. Tarokh L. Van Dongen H. P. Achermann P. (2017). Interindividual differences in the dynamics of the homeostatic process are trait-like and distinct for sleep versus wakefulness. *J. Sleep Res.* 26 171–178. 10.1111/jsr.12483 28019041

[B77] Saletin J. M. van der Helm E. Walker M. P. (2013). Structural brain correlates of human sleep oscillations. *Neuroimage* 83 658–668. 10.1016/j.neuroimage.2013.06.021 23770411 PMC4263481

[B78] Sanchez-Espinosa M. P. Atienza M. Cantero J. L. (2014). Sleep deficits in mild cognitive impairment are related to increased levels of plasma amyloid-beta and cortical thinning. *Neuroimage* 98 395–404. 10.1016/j.neuroimage.2014.05.027 24845621

[B79] Shaw M. E. Sachdev P. S. Anstey K. J. Cherbuin N. (2016). Age-related cortical thinning in cognitively healthy individuals in their 60s: the PATH Through Life study. *Neurobiol. Aging* 39 202–209. 10.1016/j.neurobiolaging.2015.12.009 26923417

[B80] Sheehan C. M. Frochen S. E. Walsemann K. M. Ailshire J. A. (2019). Are U.S. adults reporting less sleep?: findings from sleep duration trends in the National Health Interview Survey, 2004-2017. *Sleep* 42:zsy221. 10.1093/sleep/zsy221 30452725 PMC6941709

[B81] Shokri-Kojori E. Wang G. J. Wiers C. E. Demiral S. B. Guo M. Kim S. W. (2018). beta-Amyloid accumulation in the human brain after one night of sleep deprivation. *Proc. Natl. Acad. Sci. U.S.A.* 115 4483–4488. 10.1073/pnas.1721694115 29632177 PMC5924922

[B82] Suh S. Kim H. Dang-Vu T. T. Joo E. Shin C. (2016). Cortical thinning and altered cortico-cortical structural covariance of the default mode network in patients with persistent insomnia symptoms. *Sleep* 39 161–171. 10.5665/sleep.5340 26414892 PMC4678334

[B83] Thomas A. G. Dennis A. Bandettini P. A. Johansen-Berg H. (2012). The effects of aerobic activity on brain structure. *Front. Psychol.* 3:86. 10.3389/fpsyg.2012.00086 22470361 PMC3311131

[B84] Thomas C. Sadeghi N. Nayak A. Trefler A. Sarlls J. Baker C. I. (2018). Impact of time-of-day on diffusivity measures of brain tissue derived from diffusion tensor imaging. *Neuroimage* 173 25–34. 10.1016/j.neuroimage.2018.02.026 29458189

[B85] Trefler A. Sadeghi N. Thomas A. G. Pierpaoli C. Baker C. I. Thomas C. (2016). Impact of time-of-day on brain morphometric measures derived from T1-weighted magnetic resonance imaging. *Neuroimage* 133 41–52. 10.1016/j.neuroimage.2016.02.034 26921714 PMC5602560

[B86] Tremblay P. Deschamps I. (2016). Structural brain aging and speech production: a surface-based brain morphometry study. *Brain Struct. Funct.* 221 3275–3299. 10.1007/s00429-015-1100-1 26336952

[B87] Valizadeh S. A. Liem F. Mérillat S. Hänggi J. Jäncke L. (2018). Identification of individual subjects on the basis of their brain anatomical features. *Sci. Rep.* 8:5611. 10.1038/s41598-018-23696-6 29618790 PMC5884835

[B88] Van Dongen H. P. Vitellaro K. M. Dinges D. F. (2005). Individual differences in adult human sleep and wakefulness: leitmotif for a research agenda. *Sleep* 28 479–496. 10.1093/sleep/28.4.479 16171293

[B89] van Erp T. G. M. Walton E. Hibar D. P. Schmaal L. Jiang W. Glahn D. C. (2018). Cortical brain abnormalities in 4474 individuals with schizophrenia and 5098 control subjects via the Enhancing Neuro Imaging Genetics Through Meta Analysis (ENIGMA) consortium. *Biol. Psychiatry* 84 644–654. 10.1016/j.biopsych.2018.04.023 29960671 PMC6177304

[B90] Wachinger C. Golland P. Kremen W. Fischl B. Reuter M. (2015). BrainPrint: a discriminative characterization of brain morphology. *Neuroimage* 109 232–248. 10.1016/j.neuroimage.2015.01.032 25613439 PMC4340729

[B91] Wall J. Xie H. Wang X. (2017). An exploration into short-interval maintenance of adult hemispheric cortical thickness at an individual brain level. *J. Exp. Neurosci.* 11 1–14. 10.1177/1179069517733453 28989284 PMC5624352

[B92] Wulff K. Gatti S. Wettstein J. G. Foster R. G. (2010). Sleep and circadian rhythm disruption in psychiatric and neurodegenerative disease. *Nat. Rev. Neurosci.* 11 589–599. 10.1038/nrn2868 20631712

[B93] Xie H. Wall J. Wang X. (2018). Relationships in ongoing structural maintenances of the two cerebral cortices of an individual brain. *J. Exp. Neurosci.* 12 1–11. 10.1177/1179069518795875 30202210 PMC6122241

[B94] Xie L. Kang H. Xu Q. Chen M. J. Liao Y. Thiyagarajan M. (2013). Sleep drives metabolite clearance from the adult brain. *Science* 342 373–377. 10.1126/science.1241224 24136970 PMC3880190

[B95] Ye R. Touroutoglou A. Brickhouse M. Katz S. Growdon J. H. Johnson K. A. (2020). Topography of cortical thinning in the Lewy body diseases. *Neuroimage Clin.* 26:102196. 10.1016/j.nicl.2020.102196 32059167 PMC7016450

[B96] Zelinski E. L. Deibel S. H. McDonald R. J. (2014). The trouble with circadian clock dysfunction: multiple deleterious effects on the brain and body. *Neurosci. Biobehav. Rev.* 40 80–101. 10.1016/j.neubiorev.2014.01.007 24468109

[B97] Zhou W. Sailani M. R. Contrepois K. Zhou Y. Ahadi S. Leopold S. R. (2019). Longitudinal multi-omics of host-microbe dynamics in prediabetes. *Nature* 569 663–671. 10.1038/s41586-019-1236-x 31142858 PMC6666404

[B98] Zilles K. Amunts K. (2013). Individual variability is not noise. *Trends Cogn. Sci.* 17 153–155. 10.1016/j.tics.2013.02.003 23507449

